# Multigenerational effects of copper nanomaterials (CuONMs) are different of those of CuCl_2_: exposure in the soil invertebrate *Enchytraeus crypticus*

**DOI:** 10.1038/s41598-017-08911-0

**Published:** 2017-08-16

**Authors:** Rita C. Bicho, Fátima C. F. Santos, Janeck J. Scott-Fordsmand, Mónica J. B. Amorim

**Affiliations:** 1 Departamento de Biologia & CESAM, Universidade de Aveiro, 3810-193 Aveiro, Portugal; 2 Department of Bioscience, Aarhus University, Vejlsovej 25, PO BOX 314, DK-8600 Silkeborg, Denmark

## Abstract

Nanomaterials (NMs) are recommended to be tested in longer term exposures. Multigenerational (MG) studies are scarce and particularly important because effects can be transferred to the next generation. The current risk assessment framework does not include MG effects and this is a caveat for persistent materials. Here, the effects of copper NMs (CuONMs) and copper salt (CuCl_2_) were assessed in a MG exposure (4 generations in spiked soil + 2 generations in clean soil, F1 to F7 generations in total), with the standard soil model *Enchytraeus crypticus*, using relevant reproduction test effect concentrations (EC_10_, EC_50_), monitoring survival and reproduction. This represented ca. 1 year continuous exposure tests. MG effects varied with effect concentration and test materials: CuONMs caused increased toxicity for EC_10_ exposed organisms (EC_50_ did not change), and transfer to clean media reset effects, whereas CuCl_2_ reduced toxicity for EC_10_ and EC_50_, but the transfer to clean media “revived” the initial effects, i.e. close to EC_50_ levels in F7. Clearly CuONMs and CuCl_2_ cause different mechanisms of toxicity or response in the long term, not predictable based on short term or one generation studies. The present contributes for the improvement of risk assessment, adding important information for the long term exposure and effects.

## Introduction

Continuous multigenerational (MG) exposure to chemicals may induce physiological adaptations such as increased tolerance^1, 2^. Physiological adaptation processes can be related to organisms’ phenotypic plasticity. This is the ability of a genotype to display several phenotypes that can present variations in biochemistry, physiology, morphology or life traits, among others, in response to environmental changes^3^ or stressors like chemicals. Phenotypic plasticity can involve epigenetic mechanisms^3–5^ i.e. changes in gene function without altering DNA sequence and that are transferred to the next generation. Further, if gene function effects prevail in non-exposed next generations, then these are named transgenerational epigenetic effects (maternal effects)^4, 6^. Adaptation can involve change in organisms’ genetic material, where e.g. most tolerant genes can be selected^4, 7^. This change in population genetics can be preserved along generations even when the stressor is removed^4^. On the other hand, MG exposure can induce an increase in organisms’ sensitivity as shown by e.g. Yu *et al*.^8^. Other studies, where only the parental generation was exposed, show transgenerational transfer of sensitivity^9^.

For nanomaterials (NMs), long term studies are one of the key recommendations to ensure a sustainable environmental development^6, 10, 11^. However, few long term studies are available and less than a handful of MG studies are published (see Table S1 for a summary), and none in soils.

For example, for the aquatic invertebrate *Daphnia* (various species) exposure to carbon and silver NMs showed an increased sensitivity or tolerance depending on the test material and test concentrations^12, 13^. There are also studies with *Caenorhabditis elegans* ^14^ performed in simulated pore water (SPW), agar or agar media (instead of soil) studying the effects of gold NMs, the parental generation was exposed, showing increased toxicity in second (unexposed) generation, hence transgenerational effects. Additionally, Schultz *et al*.^15^ investigated the effects of AgNMs, showing increased sensitivity in second generation, and the effect remained during later generations and unexposed generations, also indicating transgenerational effects.

The effects of CuNMs in enchytraeids are well reported in the literature, assessing various endpoints, covering survival, reproduction^16, 17^, avoidance behaviour^16^, cellular energy allocation^18^ and oxidative stress^19^. Recently a novel study analysed the effects along the full life span of *E*. *crypticus* ^20^, showing that organisms exposed to CuONMs EC_50_ lived shorter than when exposed to CuCl_2_. Although, there are no MG effect studies performed for NMs.

In the present study we aimed to investigate the effects of MG exposure to copper oxide NMs (CuONMs) and copper salt (CuCl_2_), using *E*. *crypticus*, a soil model representative^21–23^. Survival and reproduction effects were assessed along four generations of continuously exposed organisms to Cu plus 2 generations in clean media, hence the transgenerational potential was also assessed (6 generations in total).

## Results

### Multigeneration (MG) test

The validity criteria from the standard test^21^ were fulfilled, i.e. for juveniles the coefficient of variation was <20%, the number of juveniles was ≥50 and adults’ mortality was ≤20%. Values for soil pH did not change significantly within concentrations and during all multigenerational tests.

**Figure 1:**
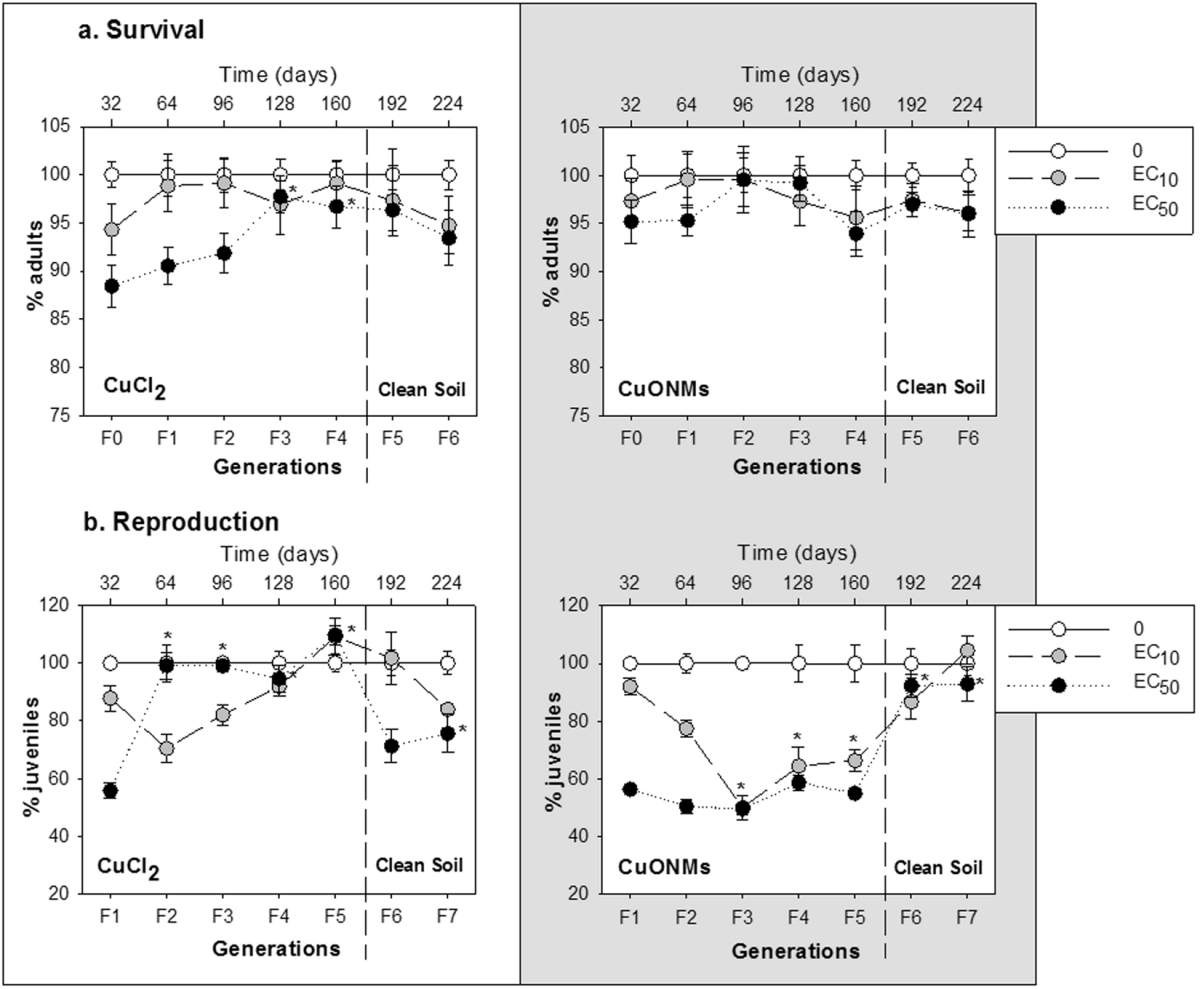
Results of the multigenerational test (MGt) for *Enchytraeus crypticus*. Exposure to the reproduction EC_10_ and EC_50_ of CuONMs and CuCl_2_ (0-500-1400 mg Cu/kg and 0-20-180 mg Cu/kg DW soil, respectively) in LUFA 2.2 soil in terms of survival (**a**) and reproduction (**b**). All values are expressed as % normalized to the control, average ± standard error (Av ± SE). *p < 0.05 (Dunnets’ between parental generation (F0/F1) and Fx).

Results of the MG test in terms of survival and reproduction can be depicted in Fig. 1.

The effect depended on the Cu materials and the test concentration. Results for reproduction show that the selected EC_10_ and EC_50_ values were approximately confirmed in F1: this corresponded for CuONMs to 8% (±2.9) and 44% (±1.5) reduction, for CuCl_2_ to 12% (±4.4) and 44% (±2.6) reduction respectively.

Results for the MG exposure with CuONMs in terms of survival showed that no significant effect occurred along generations for both exposure concentrations, which is not surprising given the sub-lethal concentrations. In terms of reproduction the EC_10_ exposure caused an increase in the toxicity followed by a levelling off while being exposed i.e. from F2 → F5: F2 = 23% decrease (F_2,21_ = 77.21, p < 0.001), F3 = 50% decrease (F_2,21_ = 99.12, p < 0.001), F4 = 36% decrease (F_2,21_ = 20.57, p < 0.001) and F5 = 34% decrease (F_2,21_ = 40.56, p < 0.001) in the number of juveniles compared with control. For the EC_50_ exposure the effect was maintained in the same level as F1 in the subsequent generations from F2 → F5. F2 = 50% decrease (F_2,21_ = 77.21, p < 0.001), F3 = 50% decrease (F_2,21_ = 99.12, p < 0.001), F4 = 41% decrease (F_2,21_ = 20.57, p < 0.001) and F5 = 45% decrease (F_2,21_ = 40.56, p < 0.001) in the number of juveniles compared with control. The transfer to clean soil (F5 → F7) showed a recovery from the effect, i.e. by F6 the number of juveniles was similar to control levels.

For CuCl_2_ the MG exposure in terms of survival showed that for EC_10_ there was a decrease in the effect from F1 → F4, with the number of adults similar to control. EC_50_ exposure caused a similar effect with values being similar to control from F3 → F4. After transfer to clean soil the organisms showed no effect, i.e. similar to control. In terms of reproduction, for EC_10_ the effect increased from F1 → F2, i.e. there was a significant reduction in the number of juveniles (30%) (F_2,21_ = 8.92, p = 0.002), after which there was a decrease from F3 → F5, showing reproduction even higher than control at F5. The transfer to clean soil at EC_10_ showed for F6 values similar to control but in F7 there was an increase in the effect similar to the F1 (EC_10_). For EC_50_, transfer to clean soil showed an increase in the effect to values close to the F1 (EC_50_), F6 = 30% reduction (F_2,21_ = 7.78, p = 0.003) and F7 = 25% reduction (F_2,21_ = 4.78, p = 0.021).

#### *In situ* characterization

Our measures show that the total Cu measured in the soil was ca. 100% of the added total concentration for both CuONMs and CuCl_2_. The total in soil solution was less than 0.07% for the controls, less than 1% of the total for CuONMs, and less than 3% for CuCl_2_. The free active Cu was less than 0.004% in controls, and less than 0.001% for both Cu forms exposure. As actual concentrations, the total Cu in soil solution for CuCl_2_ was similar or up to 3 times higher than for CuONMs at total concentrations between 200–400 mg Cu/kg. It was not possible to identify whether the Cu in the soil solution was NM or free ions, due to the small size (approx. 10 nm, table 1) of our Cu particles.

**Table 1:** Characteristics of the tested CuONMs (Source: FP7-SUN (Sustainable Nanotechnologies European Commission funded project).

Characteristics	CuONMs
Manufacturer	Plasma Chem
CAS number	1317‐38‐0
Primary size distribution (average)	3–35 (12)
Mode (1st quartile - 3rd quartile) [nm]	10 (9.2–14)
Shape	Semi-spherical
Average crystallite size [nm]	9.3
Crystallite phases (%)	Tenorite 100%
Dispersability in water: D50 [nm];	139.5 ± 4.6;
Average agglomeration number (AAN)	346
Dispersability in modified MEM: D50 [nm];	85.2 ± 2.7;
Average agglomeration number (AAN)	77
Z‐potential in UP water [mV]	+28.1 ± 0.6
Isoelectric point [pH]	10.3
Photocatalysis: photon efficiency [unitless]	1.5 × 10–4
Specific Surface Area [m2 g-1]	47.0 ± 1.7
Pore sizes [nm]	13.5 ± 1.6 (BJH) 23.0 ± 0.9 (AVG)
Surface chemistry [atomic fraction]	Cu = 0.46 ± 0.05; O = 0.47 ± 0.05 C = 0.07 ± 0.01

## Discussion

For both tested materials the MG effects were highly dependent on test concentration. For CuONM the EC_10_ exposure caused increased toxicity with a MG exposure, and by F3 the EC_10_ became similar to EC_50_ level, this being maintained in F4 and F5. On the other hand, for the EC_50_ MG exposure, the toxicity was similar from F1 to F5, hence no apparent increased tolerance or sensitivity occurred. For both ECx exposures transfer to clean soil induced full reset to no toxicity, i.e. values similar to control. The few literature results with MG with NMs show indications of either response. For example, a MG study in *D*. *magna* exposed to carbon NMs showed increased toxicity in generation F1 and its maintenance in F2^12^. In *C*. *elegans* exposed to AgNMs toxicity increased in F2 and this was maintained until F10^15^. For *C*. *elegans* transfer to clean media showed a similar toxicity as transferred, i.e. transgenerational effects, which could be related with epigenetic mechanisms^15^ although not confirmed. Li *et al*.^4^ also observed increased toxicity to mercury with MG exposures in *T*. *japonicus*, and then full recovery from effect when transferred to clean media, indicating only physiological effects rather than genetic or epigenetic changes. The same was observed to the same species on a MG exposure with Cu^24^.

For CuCl_2_ in terms of reproduction, the exposure to the EC_10_ caused an increase in toxicity for F2 and F3, and the opposite for F4 and F5, where an increased tolerance/resistance is observed after 2–3 generations. On the other hand, for the EC_50_, there was an immediate decrease in toxicity after 1 generation, i.e. from F2, this being maintained until F5 (with an actual *hormesis* like effect in F5), again suggesting an increased tolerance/resistance after F1. Similar results were observed in a previous study with *E*. *crypticus* exposed to CuCl_2_ for two generations^25^. In terms of survival, the effect also decreased after 1 exposure generation, although here to a lower extent. Such an increase in tolerance has been similarly observed in *D*. *magna* ^1^ exposed for six generations to CuCl_2_ where the reproductive output increased with concentrations of 0.5 to 100 µg Cu/L, although the decreased toxicity did not reach values as similar to control as in our study. On the other hand, the opposite has also been shown by Yu *et al*.^9^, where *C*. *elegans* was exposed for four generations to CuCl_2_ and reproduction toxicity increased for both concentrations tested (0.1 mg Cu/L and 10 mg Cu/L) along generations. Multigenerational effects of Cu investigated in the marine invertebrate *Tigriopus japonica* ^24^ for two generations, (where for each generation the offspring was transferred to clean media to evaluate recovery effects), showed similarly a decrease in toxicity for reproductive output to both tested concentrations (10 and 100 µg Cu/L), this being higher for the highest concentration (still below control); for the generations transferred to clean media, organisms presented a full recovery to same as control levels. So, not only MG effects seem dependent on the test concentration and materials, but are also species specific. Interestingly, in our MG exposed animals, the transfer to clean soil (F5 → F7) there was an increase in toxicity, with the organisms changing from F5 no effect to similar F1 effect level, like a memory effect. Results may indicate that parent organisms which are exposed to CuCl_2_ during one cycle produce offspring that developed defences or activated mechanisms to keep new homeostasis levels (Cu is an essential element); when the element is reduced in the transfer to clean media, it could be that there is now a deficit of Cu concentration for the new homeostasis levels, hence a shift in the stress type, from excess Cu to deficit. Hence, the mechanism does not necessarily seem to involve epigenetics, but rather a physiological adaptation, e.g. metallothyonein or Cu binding proteins activation. This is the first time that such a remarkable increased tolerance to Cu is shown (from EC_50_ to EC_0_ in 4 generations) and then also regained after 2 generations in clean media. This could indicate a particular plasticity of *E*. *crypticus* to CuCl_2_.

This experimental design (4 + 2) highlights that care should be taken regarding potential extrapolation of effects: one could assume that organisms exposed to Cu during 4 generations become more Cu-tolerant, although the transfer to clean soil shows that sensitivity was still embedded.

The differences observed between CuCl_2_ and CuONMs indicate different mechanisms of toxicity or response. This is also confirmed via the full life cycle test performed with *E*. *crypticus* ^26^ where effects were life stage dependent and distinct for the two Cu forms.

An interesting observation was the increased toxicity for EC_10_ exposure for both Cu forms, which did not occur for the EC_50_ exposure. Similarly, Amorim *et al*.^27^ in a MG study where *Folsomia candida* was exposed to cadmium EC_10_ and EC_50_ along more than one year, observed increased toxicity for EC_10_ exposure, leading to population extinction, although not for the EC_50_. This highlights not only the need for MG studies but also that low concentrations can have a significant and higher impact.

A much discussed issue in relation to metallic NM exposure, is whether the organisms are exposed to the NM or a dissolved part of the metal, i.e. here Cu ions. As from soil measurements, the free active Cu was less than 0.001% for both Cu forms exposure, so this does not seem to be the source for differences. On the other hand, the total Cu in soil solution for CuCl_2_ was 3 times higher than for CuONMs which could argue for a potential higher bioavailable fraction and activate mechanisms differently, although this doesn’t seem to account for all differences. Navratilova *et al*.^28^ showed that it was possible to detect larger CuONMs by Single Particle ICP-MS (the theoretical detection limit being 15 nm), but due to the interaction with soil components it was not possible to separate Cu ions bound to small natural particles from CuONMs present in the sample. However, they showed that CuONMs persisted in the nanoform (even though in the form of agglomerates) and do not completely solubilize in the presence of soil components, i.e. organic matter. Another study showed that Cu was solubilized from CuONMs in a sand matrix and also that NM remained in the media: for 500 mg Cu/kg soluble Cu was 3 mg/L just after spiking, 2.5 mg/L after 1 day and 1 mg/L after 7 and 14 days^29^. This indicates the solubility of CuONMs tending to a stabilization/equilibrium point after some time. Nevertheless, a sand matrix is a fairly basic media compared to soil.

One should be reminded that in this MG test we are limited in terms of measured endpoints to survival and reproduction, hence a refined understanding is limited. Survival and reproduction are very important and will capture other effects, but as we learned from the full life cycle test^26, 30^ including endpoints such as hatching and growth can help discriminate at what life stage the effects differs between nano and salt forms. Further studies, e.g. at the molecular level, should be performed to clarify the observed phenotypic effects, to measure the potential for epigenetics mechanisms is an obvious one.

Clearly CuONMs and CuCl_2_ caused different mechanisms of toxicity or response in the long term, in this case MG, not predictable based on short term or one generation studies. The present results can contribute for the improvement of chemicals risk assessment, adding important information for the long term exposure and effects of Cu NMs.

## Methods

### Test organisms

The test species *Enchytraeus crypticus* (Oligochaeta: Enchytraeidae) was used. *E*. *crypticus* cultures have been kept for many years at the University of Aveiro. Synchronized cultures were prepared as described in Bicho *et al*.^23^, using juveniles with 17–18 days after cocoon laying. Mature adults with well-developed clitellum are transferred to agar plates to lay cocoons; synchronized 1–2 days old cocoons are placed in new agar plates and left to grow.

### Test soil

The standard LUFA 2.2 natural soil (Speyer, Germany) was used. Soil characteristics are: pH = 5.5, organic matter = 1.77%, CEC (cation exchange capacity) = 10.1 meq/100 g, WHC (water holding capacity) = 41.8%, grain size distribution of 7.3% clay, 13.8% silt, and 78.9% sand.

### Test materials and spiking

Copper (II) chloride dihydrate (CuCl_2_·2H_2_O > 99.9% purity, Sigma-Aldrich, CAS number 10125-13-0) and copper oxide nanomaterials, CuONMs (PlasmaChem GmbH) were used. For details see Table 1.

The tested concentrations were 0-500-1400 mg Cu/kg soil (DW) for CuONMs and 0-20-180 mg Cu/kg soil (DW) for CuCl_2_. Concentrations were selected based on the reproduction effect concentrations EC_10_ and EC_50_^26^ as shown in the Table 2 for reference:

**Table 2:** Estimated effect concentration (EC) values for the Enchytraeid Reproduction Test (ERT) performed with *Enchytraeus crypticus* when exposed to CuONMs and CuCl_2_ in LUFA 2.2 soil.

	Reproduction		Survival	
	EC_10_	EC_50_	LC_10_	LC_50_
**CuONMs**	**438** (312–616)	**1377** (1157–1638)	**421** (210–631)	**2103** (1855–2352)
**CuCl**_**2**_	**18** (n.d.)	**179** (153–206)	**112** (26–198)	**303** (251–355)

Confidence intervals are shown in brackets. n.d.: not determined.

For CuONMs spiking followed the recommendations for nanomaterials^31^. Spiking was performed individually, in 5 g of soil per replicate, and mixed with the corresponding amount of the test materials (as dry powders) to the nominal concentration. The spiked soil was added to the remaining soil (35 g) and homogeneously mixed, per individual replicate, to ensure total raw amounts. Soil was allowed to equilibrate for 1 day prior test start. Moisture was adjusted to 50% of the WHC_max_. For CuCl_2_ a stock aqueous solution was done and sequentially diluted; spiking was done onto the pre-moistened soil amounts per concentration, this being homogeneously mixed and split onto replicates. Soil was freshly spiked 1 day prior each of the generations.

### *In situ* characterisation

The amount of Cu was measured in the test soil and in soil solution (for method details see Gomes *et al*.^17^) in a concurrent experiment over the test duration. In the soil the total Cu was measured (by Graphite Furnace Atomic Absorption Spectroscopy: AAS-GF) and in soil solution both the total Cu (AAS-GF) and free active form (ion-selective electrode) were measured. The CuO present as nanomaterials was not determined in the soil, due to the technical difficulties, e.g. the particle size is below the theoretical detection limit of 15 nm^28^.

### Test procedures

**Figure 2:**
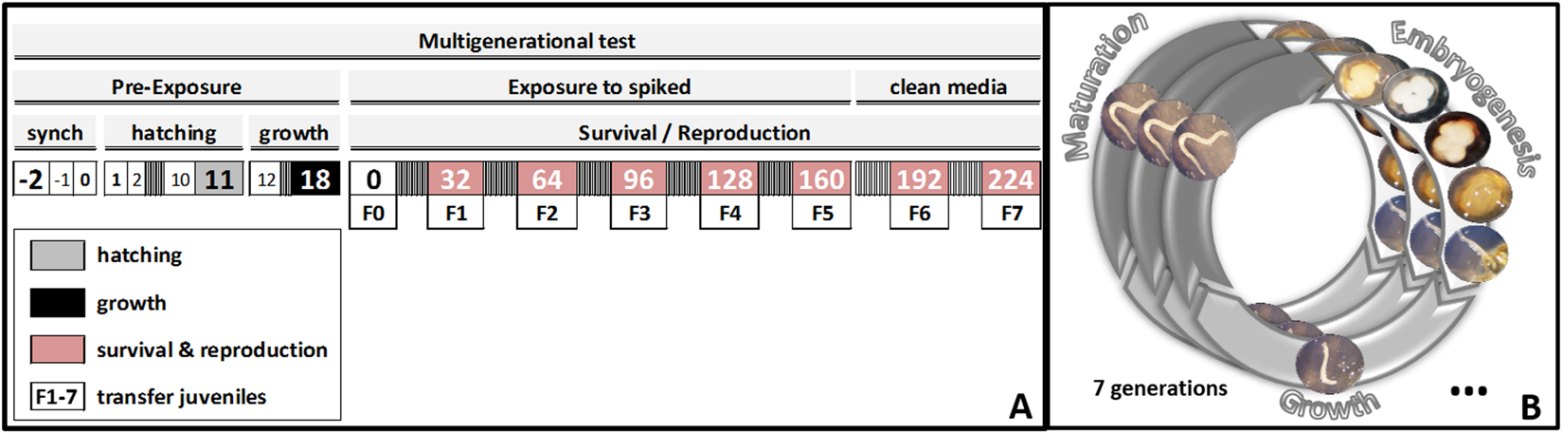
Schematic representation of *Enchytraeus crypticus* multigenerational test. The figure includes (**A**) Multigenerational test design including sampling days for synchronization and transfer between generations and (**B**) life stages from cocoon to mature adults and the following generations and associated time (days).

A first set of preliminary tests were performed to optimize sampling days, number of organisms, etc (data not shown). The final test design is illustrated in Fig. 2. Test duration per generation is 32 days, this ensures that juveniles are as large size as possible but still allowing to discriminate from adults, i.e. just before being fully grown, mature and releasing cocoons of next generation.

### Multigenerational test (MGt)

Test followed the standard guideline^21^ with adaptations as follows. Forty (40) juveniles (17–18 days’ age) per replicate were used. Organisms were collected and introduced in test vessels, containing 40 g of moist soil and food supply. For each generation tests ran during a period of 32 days at 20 °C and 16:8 h photoperiod. In total six generations were exposed, hence the total test duration was 224 days. Test design involved 4 + 2 generations, 4 in spiked soil (F0–F4) plus 2 in clean soil (F5–F6) to assess organisms’ recovery. Food and water was replenished weekly. For control and the EC_10_ six replicates per treatment were used, for the EC_50_ ten replicates were used to ensure enough number of organisms for next generations and analysis. At the end of each generation, deionized water was added to each replicate and soil was left to deposit for 20 min, after this organisms (adults and juveniles) were carefully transferred to freshly made reconstituted ISO water^32^. From each replicate adults (n = 20) and juveniles of large and medium size (n = 400) were sampled, snap-freeze and kept at −80 °C for further analysis. Juveniles of medium size (n = 40) were collected and transferred to freshly spiked test soil for another generation and so forth.

The soil and remaining organisms were counted, replicates were fixated with 96% ethanol and Bengal red (1% solution in ethanol) as described^23^.

### Data analysis

One-way analysis of variance (ANOVA) followed by Dunnett’s comparison post-hoc test (p ≤ 0.05) was used to assess differences between generation F0 and the other generations within each treatment^33^.

### Availability of data and materials’

All raw data can be made available upon request.

## Electronic supplementary material

Supplementary Table 1
