# Supplementary material for: Multigenerational effects of copper nanomaterials (CuONMs) are different of those of CuCl2: exposure in the soil invertebrate Enchytraeus crypticus
Source: Sci Rep. 2017 Aug 16;7:8457. doi: 10.1038/s41598-017-08911-0 (PMC5559477; doi:10.1038/s41598-017-08911-0)
Supplement: Supplementary file 1 — Supplementary Table 1 [file 41598_2017_8911_MOESM1_ESM.pdf]

Multigenerational effects of copper nanomaterials (CuONMs) are different of $CuCl_2$: exposure in the soil invertebrate *Enchytraeus crypticus*

Rita C. Bicho[a], Fátima C.F. Santos[a], Janeck J. Scott-Fordsmand[b], Mónica J.B. Amorim[a*]

[a]Departamento de Biologia & CESAM, Universidade de Aveiro, 3810-193 Aveiro, Portugal

[b]Department of Bioscience, Aarhus University, Vejlsovej 25, PO BOX 314, DK-8600 Silkeborg, Denmark

ritabicho@ua.pt; fatimasantos@ua.pt; jsf@bios.au.dk; mjamorim@ua.pt;

*Corresponding author at: Department of Biology & CESAM, University of Aveiro, 3810-193 Aveiro, Portugal. Tel.:+351 234 370790. Fax: +351 234 372 587. E-mail address: mjamorim@ua.pt

Table S1: Summary overview of available literature data on multigenerational exposure of invertebrate species to nanomaterials. ↑: increase, ↓: decrease; ≈: similar toxicity. Ref: reference.

Species	NM	Media	Design	Endpoint	Effect	Ref
D. magna	MWCNTs	Water	Generations: F0-F2; Exposure: parent F0	survival	↑F1	[12]
				size (length)	↓F1-F2	
	C60-βCD	Water	Generations: F0-F2; Exposure: parent	survival	↑F1	
				reproduction	↓F1	
	C60-malonate	Water	Generations: F0-F2; Exposure: parent	survival	↑F1	
				reproduction	↑F1	
				size (length)	↑F1	
	SWCNT-CONH2	Water	Generations: F0-F2; Exposure: parent	reproduction	↑F1-F2	
				size (length)	↓F1	
D. magna	Ag	Water	Generations: F0-F4; Exposure: F0-F4 + clean F3-F4	survival	↑F4	[13]
				reproduction	↓F1, ≈F2-F4, F3-F4 ≈ F2	
				population increase (*r*)	≈ F0-F4	
D. pulex	Ag	Water	Generations: F0-F4; Exposure: F0-F4	survival	↓F1,↑F2,↓F3-F4	
				reproduction	≈ F0-F4	
				population increase (*r*)	≈ F1 ↑F2 ↓F3-F4	
D. galeata	Ag	Water	Generations: F0-F4; Exposure: F0-F4	survival	↓F1,↑F2,↓F3-	
				reproduction	↑F1-F2;↓F3-	
				population increase (*r*)	↓F1,↑F2,↓F3-F4	
C. elegans	Au	Agar	Generations: F0-F4; Exposure: parent F0	survival	none	[14]
				reproduction	↓F1,↑F2,↓F4	
C. elegans	Ag	SSPW agar	Generations: F0-F10; Exposure: F0-F10 + clean F6-F10	reproduction	↑F2-F10 F6-F10 ≈ F5	[15]
